# Biochar from "Kon Tiki" flame curtain and other kilns: Effects of nutrient enrichment and kiln type on crop yield and soil chemistry

**DOI:** 10.1371/journal.pone.0176378

**Published:** 2017-04-27

**Authors:** Naba Raj Pandit, Jan Mulder, Sarah Elisabeth Hale, Hans Peter Schmidt, Gerard Cornelissen

**Affiliations:** 1 Institute for Environmental Sciences (IMV), Norwegian University of Life sciences (NMBU), Ås, Norway; 2 Nepal Agroforestry Foundation (NAF), Koteshwor, Kathmandu, Nepal; 3 Norwegian Geotechnical Institute (NGI), Oslo, Norway; 4 Ithaka Institute for Carbon Strategies, Ancienne Eglise 9, Arbaz, Switzerland; RMIT University, AUSTRALIA

## Abstract

Biochar application to soils has been investigated as a means of improving soil fertility and mitigating climate change through soil carbon sequestration. In the present work, the invasive shrub "*Eupatorium adenophorum*" was utilized as a sustainable feedstock for making biochar under different pyrolysis conditions in Nepal. Biochar was produced using several different types of kilns; four sub types of flame curtain kilns (deep-cone metal kiln, steel shielded soil pit, conical soil pit and steel small cone), brick-made traditional kiln, traditional earth-mound kiln and top lift up draft (TLUD). The resultant biochars showed consistent pH (9.1 ± 0.3), cation exchange capacities (133 ± 37 cmol_c_ kg^-1^), organic carbon contents (73.9 ± 6.4%) and surface areas (35 to 215 m^2^/g) for all kiln types. A pot trial with maize was carried out to investigate the effect on maize biomass production of the biochars made with various kilns, applied at 1% and 4% dosages. Biochars were either pretreated with hot or cold mineral nutrient enrichment (mixing with a nutrient solution before or after cooling down, respectively), or added separately from the same nutrient dosages to the soil. Significantly higher CEC *(P< 0*.*05)*, lower Al/Ca ratios *(P< 0*.*05)*, and high OC% (*P<0*.*001*) were observed for both dosages of biochar as compared to non-amended control soils. Importantly, the study showed that biochar made by flame curtain kilns resulted in the same agronomic effect as biochar made by the other kilns (*P > 0*.*05*). At a dosage of 1% biochar, the hot nutrient-enriched biochar led to significant increases of 153% in above ground biomass production compared to cold nutrient-enriched biochar and 209% compared to biochar added separately from the nutrients. Liquid nutrient enhancement of biochar thus improved fertilizer effectiveness compared to separate application of biochar and fertilizer.

## Introduction

Biochar (BC) is the carbon-rich material produced by the pyrolysis of biomass i.e. heating in the partial or complete absence of oxygen [1]. Biochar is highly recalcitrant in nature unlike other forms of soil organic matter (SOM). Thus, biochar amendment to soils acts as a carbon sequestration technique which can also enhance soil fertility [1–3]. Agronomic benefits of biochar-amended soils can be the result of improved soil physical properties (bulk density, porosity, water holding capacity, permeability, aggregation), biological properties (improved environment for microbial populations such as mycorrhizae) and chemical properties (pH, CEC and nutrient retention capacity) [4–11].

Various pyrolysis technologies and various feedstocks can be used to produce biochar. This may result in a large variation in resulting biochar properties [12,13] which in turn may affect biochar effectiveness for increasing soil fertility [14,15]. Low temperature pyrolysis (300–500°C) has shown increased biochar yield and carbon content whereas high temperature pyrolysis (>500°C) has revealed lower biochar yield and higher surface area with increased adsorption capacities for various compounds [16]. Research on the effect of pyrolysis technology on agronomic biochar quality has up until now been scarce. Under rural (sub)-tropical conditions, biochar has mostly been produced with medium-sized traditional kilns made of bricks or simple earth mound heaps, improved retort kilns [17,18] or top-lit up-draft (TLUD) pyrolysis units [19]. Traditional kilns can be operated using all kinds of mixed biomass feedstocks. However, pyrolysis gases such as methane (CH_4_), carbon monoxide (CO) and aerosols (PM 2.5 and PM 10) are released untreated, and this leads to greenhouse gas emissions, pollutant emissions and loss of energy [20]. Improved retort kilns have features to recirculate the produced syngases into the combustion chamber, resulting in up to 75% less toxic and greenhouse gas emissions as well as higher conversion efficiency (40–50%) compared to traditional brick kiln, due to less losses of energy-rich molecules [21]. On the other hand, improved retort kilns are more costly, difficult to operate and often consume a lot of start-up biomass materials [18]. TLUD kilns burn feedstock cleanly, thereby reducing gas emissions, as the syngases are combusted largely in the flame front. If used indoors this reduces negative health impacts [22]. There are some limitations with using relatively small TLUDs as they produce so little biochar (around 300 g per run) that they are mainly useful for small-scale kitchen gardening [20]. Larger TLUDs, while generating more biochar, require significant investments and expertise in order to be operated successfully.

To circumvent such challenges, the flame curtain, open pit "Kon-Tiki" kiln was recently developed [23]. It follows the principle of pyrolyzing biomass layer after layer in an open, conically built metal kiln that is easy to operate, fast, and results in low greenhouse gas emissions [20]. It thus allows biochar production in relatively large quantities (700 to 850 L volume biochar in 4–5 hours) [20–23]. The flame curtain kiln can even be operated as a simple conically shaped hole in the ground, leading to the same low emissions and similar biochar quality as the metal version, but essentially without any cost apart from the few hours of labour required to dig and prepare the soil pit [20].

Most studies on weathered soils have shown significant positive effects of biochar application on crop production; however, other studies have not shown any significant or even negative effects of biochar on crop yield [24,25]. Some examples from tropical countries on mostly acidic and weathered soils include the following. Radish yield increased significantly in biochar amended soils blended with mineral N fertilizers in pot trials, emphasizing the role of biochar in improving nitrogen use efficiency [2]. Moreover, conservation farming practice carried out with 4 tons/ha of biochar in a maize field in Kaoma, Zambia characterized by sandy acidic soils result in strong increases (0.9 ± 0.1 t ha^-^ without biochar to 3.8 ± 0.5 t ha^-^ with biochar) in crop yield [26]. Furthermore, application of biochar at 10 t ha^-1^ along with NPK mineral fertilizers (50g m^-2^) in maize, cowpea and peanut field showed an increase of 322%, 300% and 200% respectively compared with control plot (without biochar and NPK) in South Sumatra, Indonesia [7]. In contrast, field application of biochar did not show agronomic effects at four sites out of six in Zambia [26]. In seven field trials on five working farms in the UK, [27] observed positive yield effects in three trials, no effects in three trials and negative yield effects in one trial.

Recently, techniques for biochar nutrient enrichment, i.e. mixing nutrients with biochar before addition to the soil, have resulted in some promising increases in crop yield. Biochar enriched with cattle urine and amended to soil in Dhading, Nepal, increased the yield of pumpkin to 82.6 t ha^-1^ [28], more than 300% higher than that with only urine and 85% higher than the yield with the same amount of biochar without urine added. In another study, biochar enriched with compost nutrients by co-composting in the presence of biochar, was added to sandy soils and increased the yield of Chenopodium quinoa by 300% compared to non-enriched biochar treatments in the presence and absence of compost [29]. Biochar nutrient enrichment is probably effective due to penetration of nutrients in biochar micro- and nanopores. The pores of carbonaceous sorbents such as biochar are so narrow that water movement is restricted and an ice-like water structure is formed [30]. Earlier work has provided evidence of a relation between organic compound sorption and the nanopore volume of such matrices [30] and it is possible that a similar phenomenon could occur for nutrients in biochar. Nutrient addition to biochar has thus shown to be a promising method to enrich the biochar and render it a slow-release fertilizer. However, systematic studies on the optimal way to carry out such nutrient enrichments are lacking.

This is the first study to directly compare the agronomic effect of biochar produced from different kiln types and enriched in different ways (enriched hot biochar and enriched cooled-down biochar, as compared to non-enriched biochar where the same amount of nutrients was added separately). The study was carried out using a pot trial design in Nepal using a woody shrub as biochar feedstock. "*Eupatorium adenophorum*" is a promising feedstock as it is a naturally regenerating, ubiquitous, invasive woody forest shrub species locally named "Banmara" (forest killer) that is about 1–2 m high and stems up to 2 cm thick [31]. In this way, waste from an invasive species can be turned into a valuable resource for agronomic production and carbon sequestration. Biochar produced from Eupatorium feedstock has been found to meet all the requirements for premium quality based on European Biochar certificate [20]. In Nepal, average landholding size is very small and the soils can be acidic, exhibiting lower levels of C, N, P and exchangeable bases [32]. Overall, this study tested the following hypotheses: (1) Biochar produced from various kilns with different pyrolysis conditions exhibits different crop yield effects depending on kiln type, and (2) Nutrient enrichment improves the agronomic effect of biochar thereby increasing the maize biomass production.

## Materials and methods

### Biochar

Biochar (BC) was produced using several different types of kilns; flame curtain kilns (four sub types: deep-cone metal kiln, steel-shielded soil pit, conical soil pit and steel small cone kiln), brick-made traditional kilns, traditional earth-mound kilns, and TLUD kilns. Photographs of each of these production methods are shown in the supporting information (Image A in S1 File) along with a description and principle of their operation (Description A in S1 File). The feedstock used for the generation of biochar was the woody shrub Eupatorium, which was collected from forests close to the site of pot trials at Matatirtha, Kathmandu, Nepal (N 27° 41' 51", E 85° 14' 0", altitude 1520 m). Stems were 1–2 cm thick. Eupatorium had 25% moisture content at the time of pyrolysis [20]. Elemental analysis of the Eupatorium was carried out using an *EuroEA Elemental Analyzer* and showed that the biomass contained 42.9% C, 1.4% H and 1.5% N. For the flame curtain kilns, Eupatorium was subjected to a maximal pyrolysis temperature of around 600°C just below the flame curtain, as measured by an Impex digital thermometer with a 60-cm temperature-resistant sensor pin [20,23] cooling down to 200–400°C as the pyrolyzing biomass was getting further and further down below the flame curtain upon the layer-by-layer addition of new feedstocks. Pyrolysis temperatures for the other kilns were lower, around 400 to 500°C before final quenching with soil or water [17]. Following the pyrolysis process which took place over a period of around 2 hours per batch, biochars produced from the deep-cone metal flame curtain kiln, steel small cone, TLUD and brick kiln were quenched or snuffed with water whereas biochar produced from the steel shielded soil pit and conical soil pit flame curtain kilns were snuffed with soil (Image A in S1 File). Weight and volume of the biochar were measured after water snuffing and soil snuffing.

### Biochar nutrient enrichment

Biochar was nutrient-enriched using two methods, namely hot and cold nutrient enrichment. Hot and cold nutrient enrichment refers to hot and cooled-down biochar, respectively, that were enriched with mineral fertilizers (NPK) added dissolved in water. Hot nutrient enrichment was carried out by pouring hot (200 to 400 C) biochar at the rate of 30 g and 120 g (equivalent to 1% (20 t ha^-1^) and 4% (80 t ha^-1^ biochar respectively) in 1 L dissolved nutrients in a bucket. For both biochar rates, all biochar was submerged, however, biochar for the 1% amendments was enriched in a thinner slurry (higher liquid to solid ratio) than the biochar added at a 4% rate. During hot nutrient enrichment, the biochar was cooled down from 200–400 ^0^C to < 40 ^0^C upon contact with the nutrient solution. The nutrient solution contained urea, di-ammonium phosphate (DAP) and potash as the source of nitrogen (N), phosphorous (P) and potassium (K) respectively. Urea, DAP and potash was used at the rate of 5.11 g pot^-1^, 2.34 g pot^-1^ and 1.8 g pot^-1^ which is equivalent to 2.7g pot^-1^ N, 1.08 g pot^-1^ P and 1.08 g pot^-1^ K. The lukewarm mixture in the bucket was then stirred thoroughly for 10 minutes to ensure the biochar was well mixed with the solution. Cold nutrient enrichment was carried out using a similar method with the same volume of water and amount of NPK but adding biochar that was water quenched and cooled down beforehand. After enrichment, the bucket was sealed and the biochar allowed to rest for 10 days. The liquid remaining that was not absorbed by the biochar was later added to the respective treatment pot to ensure the same fertilizer dose addition to each respective pot.

### Soil

The soil used for the pot trial was taken from a field at Rasuwa farmland (27^0^, 59,479' N and 85^0^, 11.987' E, altitude 1365m). The study was conducted on private farmland. No specific permission apart from that from the farmer was required for these locations to take the composite soil sample. The exiting field trials in Rasuwa did not involve endangered or protected species. The soil was collected from 0–30 cm depth and was well homogenized by repeated shoveling. The soil was an inceptisol (order) having low soil pH of 4.5 and base saturation of less than 50% [33].

### Pot trial

A pot trial was carried out in order to investigate the effect of different biochars, produced using different methods and enriched in different ways (hot mineral nutrient-enriched, cold mineral nutrient-enriched and non-enriched biochar) had on soil characteristics and crop production. The pot trial was carried out in June-July 2015 in a greenhouse located in Matatirtha, Kathmandu, Nepal. The average daily temperature for the time period when the pot trial was carried out were 22^0^ C (minimum 15 ^0^ C and maximum 29 ^0^ C). However, temperatures in the greenhouse were higher than those values (minimum 20 ^0^ C and maximum 49 ^0^ C). Nursery plant pots (25cm top diameter and 25 cm height) were filled with 3 kg dry soil. Biochar (dry or slurry, dependent on treatment) was added to the pots at two different doses; 1 and 4% biochar (approximately 20 t and 80 t biochar ha^-1^) based on dry soil and biochar weight and were mixed until completely homogeneous.

Seven different kiln types (7 levels), three mineral nutrient enrichment techniques (hot mineral nutrient enrichment, cold mineral nutrient enrichment and non-enrichment) each with 1% and 4% biochar dosages (6 levels) and their interaction with kiln type and nutrient enrichment techniques along with two controls illustrated 21 treatments/levels (N = 86) in total (Table 1). For biochar produced from flame curtain deep cone metal kilns and traditional brick kilns, two dosages of biochar (1% and 4% biochar) were used for hot mineral nutrient enrichment, cold mineral nutrient enrichment and non-enrichment (biochar separately added to the soil), leading to a total of 12 treatments for these production methods. For the TLUD produced biochar, the same two dosages of biochar were used, but the biochar was not enriched. For the conical soil pit, steel shielded soil pit and traditional kiln production methods, only one dosage (4%) of biochar (not enriched) was used (Table 1). In addition to these biochar additions (Table 1), two control treatments i.e. control (C1) without biochar and without NPK (non-fertilized control, n = 4) and a control (C2) with only mineral fertilizer (fertilized control, n = 5) were also used.

**Table 1:** Treatments to test biochar quality variations with (i) kiln type, and (ii) nutrient enrichment type and iii) interaction of kiln type and nutrient enrichment type biochar. These biochar type consists of 19 levels (N = 77) with two additional control treatments C1 and C2 (N = 9) where all biochar amended treatments (19 levels, N = 77) were compared with these control treatments (N = 9). The numbers T1 to T21 correspond to different treatments number with its respective replications (n = 3, 4 or 5, N = 86) in parentheses.

		Nutrient enrichment type					
		1% BC hot mineral nutrient enrichment	4% BC hot mineral nutrient enrichment	1% BC cold mineral nutrient enrichment	4% BC cold mineral nutrient enrichment	1% BC non-enriched	4% BC non-enriched
**Kiln type**	Traditional brick kiln	T1 (n = 5)	T2 (n = 4)	T3 (n = 5)	T4 (n = 3)	T5 (n = 4)	T6 (n = 3)
	Deep cone metal kiln	T7 (n = 5)	T8 (n = 4)	T9 (n = 4)	T10 (n = 5)	T11 (n = 4)	T12 (n = 3)
	Small cone kiln	-	-	-	-	T13 (n = 5)	T14 (n = 4)
	TLUD (top lift up draft)	-	-	-	-	T15 (n = 4)	T16 (n = 4)
	Conical soil pit	-	-	-	-	-	T17 (n = 3)
	Steel shielded soil pit	-	-	-	-	-	T18 (n = 3)
	Traditional earth mound	-	-	-	-	-	T19 (n = 5)
**Control**	Non-fertilized control (C1)	T20 (n = 4)					
	Fertilized control (C2)	T21 (n = 5)					

Two maize seeds were initially sown 2 cm below the soil surface in each pot. Upon germination and emergence of two leaves (after 12 days), the smaller plant, selected based on visual observation, was removed from the pot to leave one plant for the experimental duration. Each pot was watered daily with 0.7 L (corresponding to 20 mm rainfall) water. Pots were arranged in randomized complete block design (RCBD) comprising five blocks/replications. Pots in each block were rotated at an 8-day interval to ensure the homogeneity of the treatments. Weeding was carried out 20 d (1^st^ weeding) and 35 d (2^nd^ weeding) after sowing.

### Biochar, soil and maize plant analyses

Maize plants were harvested after 50 d and were separated into above ground biomass (AGB which comprised the shoot) and below ground biomass (BGB which comprised the root), just above the brace roots. Both AGB and BGB fresh weight were measured immediately after harvesting. Roots were washed carefully with clean water. Plant biomass (AGB and BGB) was oven dried at 70 ^0^C for 24 hours for dry weight analyses.

Soil samples were collected after harvesting of maize plants. Soil from all individual replicate pots was collected to make a composite sample for each of the 21 treatments. Soil analyses were conducted both prior and after the amendment of the biochar, i.e., in the presence and absence of biochar. The biochar-amended soils were analyzed after the experiment (various biochar amended treatment soils). Soil samples were oven dried at 40 ^0^C for three days and passed through a 2mm sieve and ground (< 2mm) prior to analysis. Sieved samples were used for determining pH and cation exchange capacity (CEC) and ground samples were used for total carbon, nitrogen and hydrogen (CHN) analysis. Soil pH was measured in both 0.01M CaCl_2_ and in water (soil: solution ratio of 1:2.5 in volume basis) using an Orion 1 Ross pH electrode. Total CHN was measured by elemental analysis using an *EuroEA Elemental Analyzer*. For CEC measurement, NH_4_NO_3_ extractable cations were extracted by adding 25ml 1M NH_4_NO_3_ to 3g soil, gently shaken and kept overnight. The suspension was transferred to 250ml volumetric flask through the funnel with washed blue ribbon filters (Whatman 589/3) until 250ml was collected. 15ml of 1M NH_4_NO_3_ extracted solution was poured in 15ml ICP tubes (Inductively Coupled Plasma) to measure the individual exchangeable cations (Ca^2+^, Mg^2+^, Na^+^, K^+^ and Al^3+^). For H^+^ determinations, the 1M NH_4_NO_3_ extraction solutions (20ml) were titrated with 0.05 M NaOH.

Biochar generated from the different kilns was collected after production. Biochar samples were treated in the same way as soil samples and analyzed for pH, CEC and total CHN. BET surface area was determined by N_2_ adsorption at 77 K using an automated surface area analyzer. The samples were outgassed by heating at 110o C under a flow of ultrahigh purity helium at 10 cm^3^min^-1^ for 16 to 24 h prior to analysis. Isotherm data were recorded at partial N_2_ pressures of 0.03 to 0.7 atm. The apparent surface areas of samples were obtained from the statistical monolayer capacities of N2 from the BET plots [34]. Because of the risk of N losses as NH_3_, the concentration of N absorbed to the char was measured in the study. Since P and K through volatilization can be ruled out, these nutrients were not analyzed in the enriched chars. For mineral N (N_min_) analysis, (NO_3_^-^ and NH_4_^+^) in char, biochar sample operating hot mineral nutrient enrichment was collected. N_min_ analysis was performed through standard 2M KCl extraction methods.

### Statistical analysis

Data were statistically analyzed using R software (R version 3.2.2, R commander 2.2–1) and excel. Data normality was checked prior to performing linear model ANOVA analysis. Two factor linear ANOVA model was used to explore the effect of kiln type's biochar (7 levels) and mineral nutrient enrichment techniques (hot, cold and non-enriched) including both dosages of biochar (6 levels) and their interactions (19 levels) on maize biomass yield (dry AGB, height and node diameter) (Table 1). Biochar produced from different kiln types and three different mineral nutrient enrichment types (19 levels) were compared with non-fertilized and fertilized control treatments (2 levels) via one way ANOVA. Significant effect observed in the ANOVA were further explored through Post Hoc Tukey test to compare all the treatment means and their significance against each other on maize biomass production. Soil samples were pooled per treatment for statistical analysis where the effect of biochar amended soils i.e. 1% biochar (n = 8) and 4% biochar (n = 11) on soil pH, CEC, Ca/Al and total CHN content were compared with non-fertilized and fertilized control soils.

## Results

### Biochar yield and properties

As earlier reported [20], average biochar yields from Eupatorium feedstock on dry weight basis and carbon basis were 19.5 ± 5.0% and 40.2 ± 10.1%, respectively (Table A in S1 File). These numbers were in the same order of magnitude as those for biochar from various other kiln techniques at various pyrolysis temperatures [35–38].

Chemical analysis of biochar samples showed a consistent pH of 10.12 ± 0.19 (H_2_O extraction) and 9.11 ± 0.27 (CaCl_2_ solution), which showed that variation in pyrolysis temperature between flame curtain kilns and traditional methods did not influence the pH of biochar. On average, biochars produced from different kilns all had relatively high CECs of 133.3 ± 37.2 cmol_c_ kg^-1^. Total C, H and N content of biochar samples produced from different kiln types were 73.9 ± 6.4%, 1.81 ± 0.43% and 0.74 ± 0.16% respectively. Average surface areas (SA) of biochar samples were 97 m^2^/g, ranging from 35.4 to 215 m^2^/g (Table A in S1 File). These results show that alkaline biochar with high CEC, C content and SA was produced independent of the various production methods tested in this work (the novel flame curtain, TLUD, traditional brick and earth-mound kiln).

N analysis (NO_3_—N) of hot nutrient-enriched biochar showed 1.08 ± 0.12 mg NO_3_^-^ kg^-1^ biochar and 0.81 ± 0.02 mg NO_3_^-^ kg^-1^ biochar for the biochar added at 1% and 4% respectively (Table B in S1 File). Similarly, 313 ± 5.77 mg NH_4_^+^ kg^-1^ biochar and 120 mg NH_4_^+^ kg^-1^ biochar was observed for hot nutrient-enriched biochar to be added at 1% and 4% dosages, respectively (Table B in S1 File). These N_min_ contents were likely underestimated as only one singular KCl extraction was done while Kammann et al [29] and Haider et al [39] have recently demonstrated that serial KCl extractions of biochar may lead to significant higher N_min_ quantities captured by biochar. Total N contents for hot nutrient-enriched biochar were 4.3% and 2.5%, respectively, for the biochar to be added at 1% and 4% dosages. Based on the amount of nutrients in the enrichment solution, it could be calculated that between 50 and 100% of the added N was retrieved in the biochar (Table B in S1 File).

### Biochar effect on soil properties

**Fig 1:**
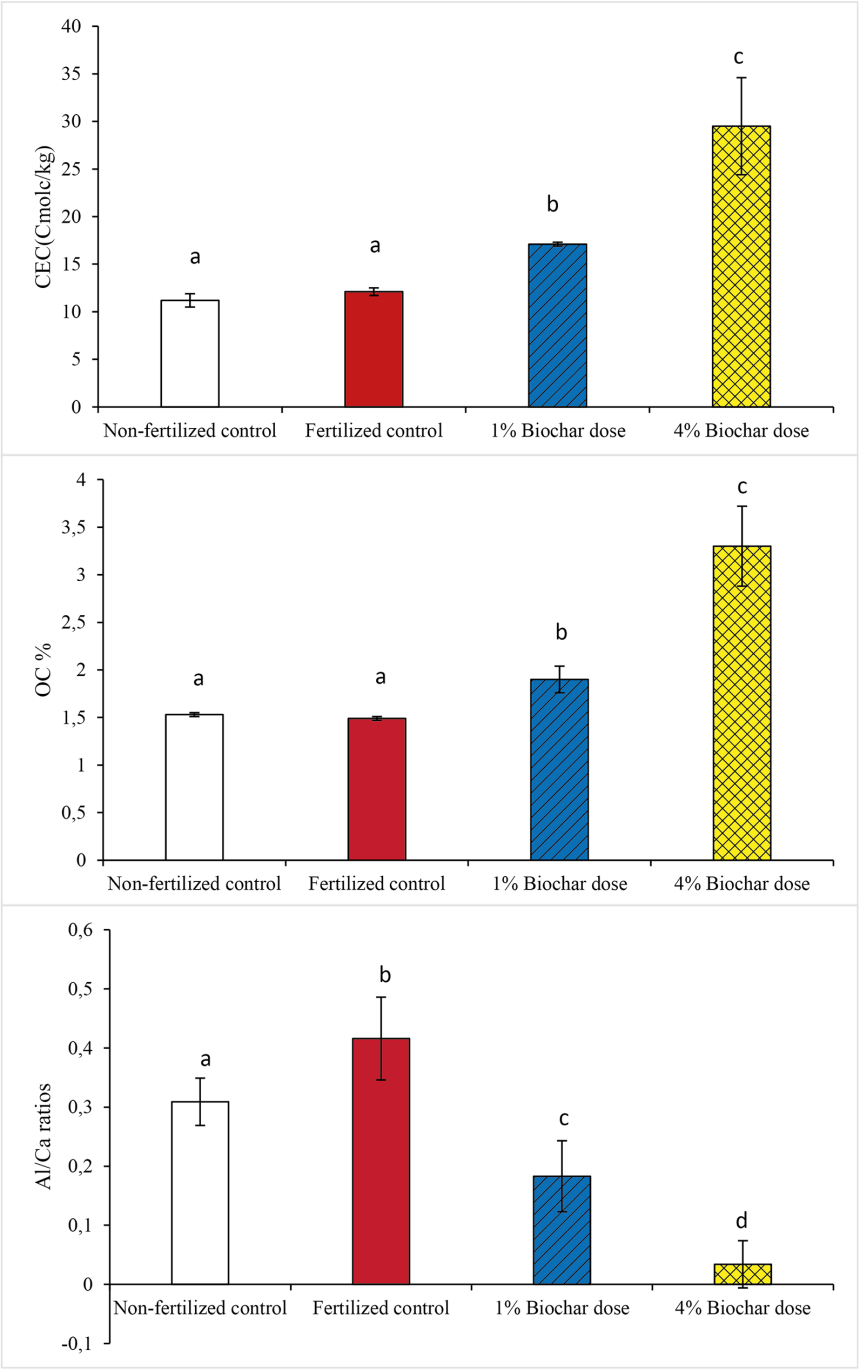
Effect of biochar dosage (1% and 4% biochar) on soil CEC, OC% and Al/Ca ratios. Biochar produced from different kiln either hot or cold mineral nutrient enrichment or non-enriched were pooled together for the statistical analysis to assess the effect of biochar dosages (1% and 4% biochar) and non/fertilized control (without biochar) on soil properties. Average CEC, OC% and Al/Ca ratio plotted on y-axis and 1% biochar dose (n = 9), 4% biochar dose (n = 11) and fertilized and non-fertilized control (n = 2) treatments were plotted against x-axis. Significance codes (a, b….) were provided based on t-test at 0.05 level of significance.

The tested Rasuwa soil was sandy and acidic with low pH (4.5), CEC (12.3 cmolc/kg) and organic carbon (OC; 1.5%). Biochar-amended soils showed increased average pH (4.84 ± 0.50) compared with the fertilized control soil (4.30 ± 0.02) (Table C in S1 File). Average Al/Ca ratios after addition of 1% biochar dose (0.18 ± 0.06) and 4% biochar dose (0.03 ± 0.04) were significantly lower (*p < 0*.*05*) than those of non-fertilized (0.30 ± 0.04) and fertilized (0.36 ± 0.08) control soils (Fig 1). Absolute exchangeable Al (III) contents of the unamended soils (0.8 to 1.0 cmol_c_/kg) were within the range where toxic Al effects on plant roots can be expected [6,26]. Average CEC after amendment with 1% and 4% biochar dosages were 17.1 ± 0.1 cmol_c_/kg and 29.5 ± 5.1 cmol_c_/kg, respectively, significantly higher *(P< 0*.*05)* than those of non-fertilized (11.2 ± 0.7 cmolc/kg) and fertilized (12.1 ± 0.4 cmolc/kg) control soils (Fig 1, Table C in S1 File). The increase in CEC was higher than expected on the basis of additivity, which is probably caused by the pH effect of biochar, resulting in an increase in CEC measured by extraction with non-buffered NH_4_NO_3_ solution. Also soil organic carbon (SOC) contents with the 1% biochar dose (1.9 ± 0.1%) and the 4% biochar dose (3.3 ± 0.4%) were significantly higher (*P<0*.*001*) than those of control treatments (1.5 ± 0.1%) (Fig 1). However, addition of biochar (70% C) for 1% and 4% biochar dosages to soil containing 1.5% SOC should have resulted in around 2.2% SOC and 4.3% SOC on the basis of pure additivity, which was higher than the measured values of 1.9% and 3.3% SOC, respectively. Hence, in contrast to CEC, the amount of SOC in the biochar-amended soil was less than expected on the amount of C added via the biochar. There were no significant variations between biochar properties arising from the use of different kilns. Thus, the improved soil chemical properties were the result of biochar addition irrespective of pyrolysis technique.

### Maize biomass production in biochar vs. non-biochar soils

**Fig 2:**
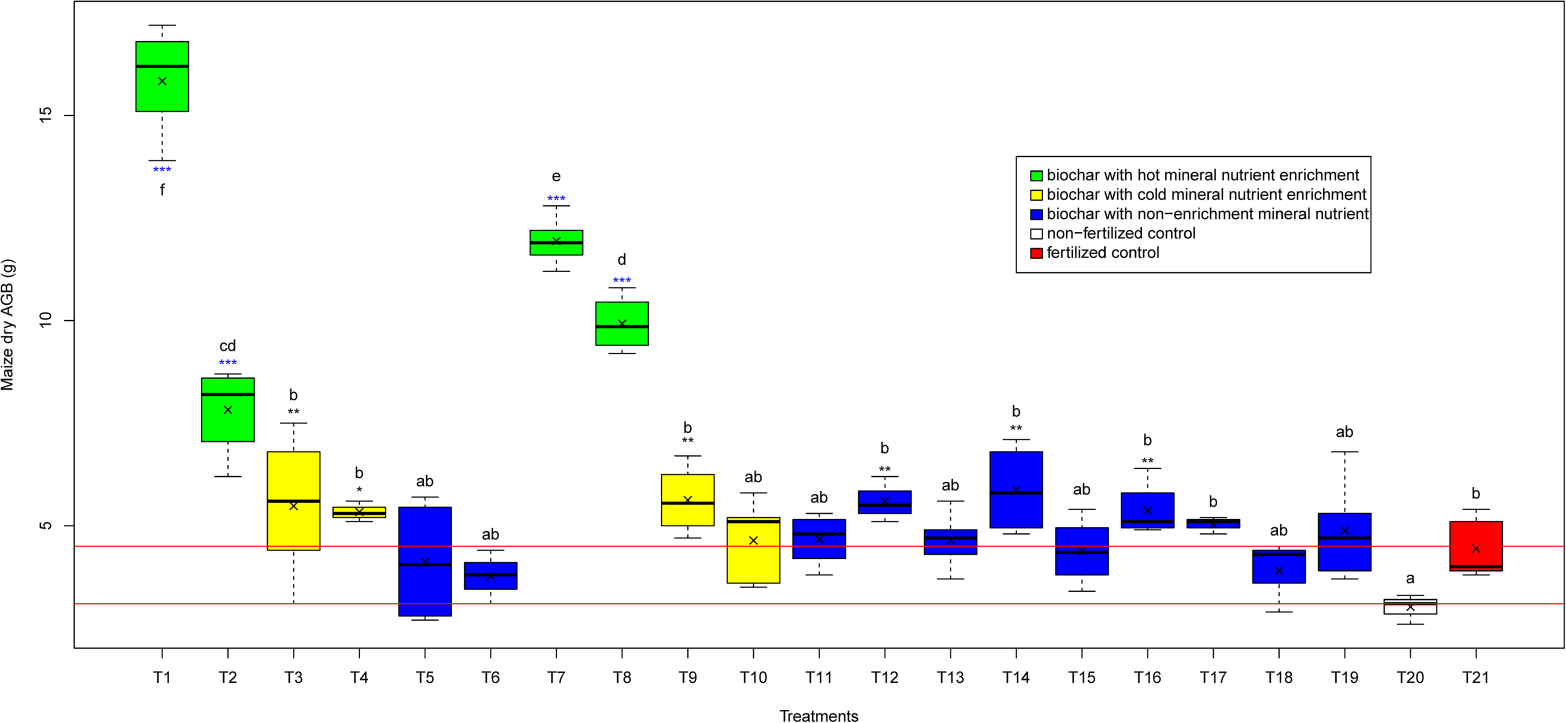
Effect of biochar amended soils produced from different kiln and enriched and non-enriched in various ways with 1 and 4% biochar dosages (19 levels; T1, T2…T19) vs fertilized and non-fertilized control treatments (2 levels; T20 and T21) on maize above ground biomass. Description of treatments (T1, T2…T21) is mentioned in the Table 1 and Table D in S1 File. Sign (x) in the middle of the box plot refer to the average maize AGB of each treatments. Asterisk (*) at the top of the box plot denotes the significant difference between biochar treatments over control (C1/T20 for non-fertilized control and C2/T21 for fertilized control) treatments (*** < 0.001, ** <0.01 and * <0.05 significance). Blue color asterisk (*) represents significance level for both non-fertilized (no color fill) and fertilized control (red color box plot) whereas black color (*) only for non-fertilized control (C1/T20). Different letters above box plot (a, b, c) represent significant differences between the treatments (T1 to T21).

**Fig 3:**
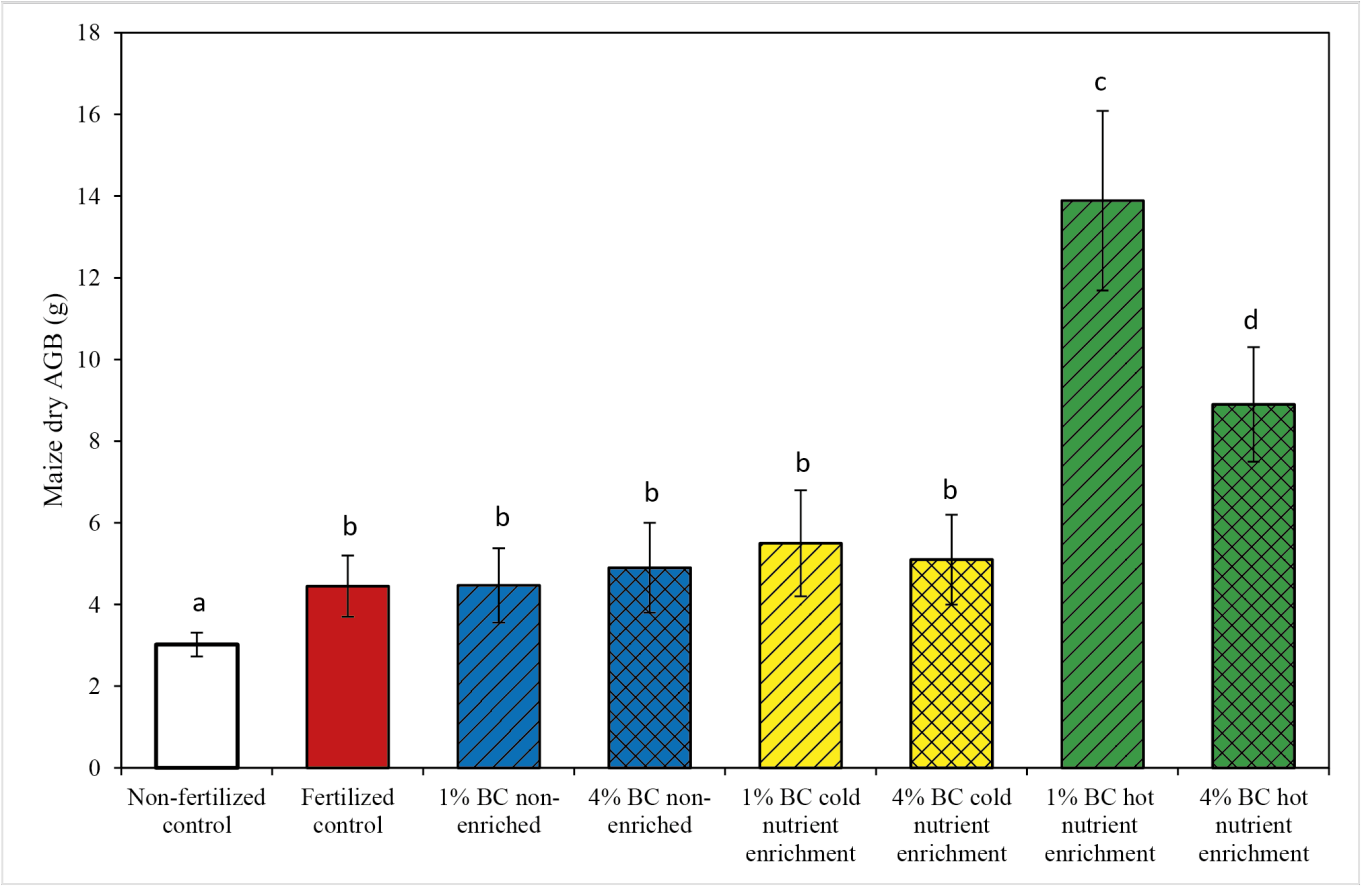
Effect of hot and cold mineral nutrient enrichment and non-enrichment type's biochar on maize dry AGB yield (g). Maize dry AGB (g) is plotted as a function of mineral nutrient enrichment technique, along with two controls. Different letters above the bars (a, b, c) represent significant differences between mineral nutrient enrichment types and controls.

Before comparing the effect of the different types of biochar production method and the nutrient enrichment techniques on crop yield, we present the results of the overall effect of biochar amendment on maize biomass production. All biochar amended treatments (21 levels, N = 86) revealed significant effect (*P<0*.*0001)* on maize biomass production. This was expressed by both maize above ground biomass (AGB) (*P<0*.*0001*), maize height (*P<0*.*0001*) and, to a lesser extent, maize node diameter (*P<0*.*0001*). Among all biochar amended soils, AGB production increased most with 1% hot mineral nutrient enriched biochar produced from traditional brick kiln (+ 248%) and produced from deep cone metal kiln (+168%), respectively, compared with fertilized control (Fig 2). Similarly, 4% biochar produced from traditional brick kiln and deep cone metal kiln encompassing hot mineral nutrient enrichment increased AGB production to 176% and 223%, respectively, of the values of fertilized control pots (Fig 2). Average maize dry AGB production per pot as a main effect of 1% biochar dosage and 4% biochar dosage increased to 165% and 139% (*P < 0*.*001*) respectively of the values of the fertilized control soils without biochar (Fig A in S1 File). Similar trends were found for maize height and node diameter. Lowest maize biomass production (3.02 ± 0.29 g pot^-1^) was observed for non-fertilized control compared with biochar amended and fertilized control treatments (Fig 2, Fig 3, Table D in S1 File).

### Effect of biochar made with different kiln types on maize biomass

Biochar produced from seven different kiln types did not show significant variation in maize biomass production (dry AGB, height and node diameter) (Table 2). When the various kiln methods were compared to each other, maize AGB production did not show significant variation for both non-enriched biochar (produced from all seven different kiln types tested) and nutrient enriched biochar (produced from traditional brick kiln and flame curtain deep cone metal kiln; the only kiln types for which biochar enrichment was tested) (Fig D in S1 File). Thus, the agronomic effect of the flame curtain kiln biochar was similar to that of the other kiln types. On average for all kiln types, maize AGB, height and node diameter for non-enriched biochar were 4.7 ± 0.7 g, 54.7 ± 6.4 cm and 2.0 ± 0.3 cm respectively (Table D panel A in S1 File). On average for both kiln types (flame curtain and traditional brick kiln), nutrient-enriched biochar showed average maize dry AGB, height and node diameter of 8.6 ± 4.0 g, 78.5 ± 26.5 cm and 3.0 ± 0.8 cm respectively (Table E panel B in S1 File). Hence, biochar generation technique had no effect on maize biomass production, but nutrient enrichment had.

**Table 2:** Statistical analysis of two factor ANOVA (kiln type and mineral nutrient enrichment type's biochar) on maize biomass yield (N = 77). The table output corresponds to Fig D in S1 File for the effect of kiln type biochar, Fig 3 for nutrient enrichment type biochar and Fig 2 and Fig G in S1 File for the interaction between kiln type and nutrient enrichment type biochar on maize above ground biomass production (gm).

	Maize dry AGB (g)		Maize height (cm)		Maize node diameter (cm)	
Factor	*f-value*	*P*	*f-value*	*P*	*f-value*	*P*
Kiln type	*1*.*2*	*> 0*.*1*	*1*.*4*	*> 0*.*1*	*2*.*3*	*> 0*.*05*
Nutrient enrichment	*123*.*4*	*< 0*.*0001*	*104*.*5*	*<0*.*0001*	*24*.*9*	*< 0*.*0001*
Kiln type and nutrient enrichment type	*7*.*5*	*< 0*.*001*	*3*.*5*	*< 0*.*01*	*1*.*3*	*> 0*.*01*

### Effect of nutrient enrichment of biochar on maize biomass

Nutrient enrichment showed significant effects (*P<0*.*0001*) on maize biomass production (Table 2). Biochar hot nutrient enrichment at 1% dosage showed increases in average maize AGB of +153% and +209% of the values observed for cold nutrient-enriched and non-enriched biochar respectively, at the same dosage of biochar and nutrients, the nutrients having been added separately for the non-enriched biochars (Fig 3). Similarly, the higher 4%-dosage hot nutrient enriched biochar showed higher (*P<0*.*001*) average AGB than the 4% non-enriched (+82%) and cold-enriched biochars (+62%)(Fig 3). The study also showed that 1% hot nutrient enriched biochar amendment gave significantly higher maize biomass (*P<0*.*0001*) than all of the 4% biochar treatments (hot nutrient enrichment, cold nutrient enrichment and non-enriched) (Fig 3). Similar trends were observed for maize height and maize node diameter (Figs E and F in S1 File). Overall, both dosages of biochar treated via hot nutrient enrichment showed significantly stronger effects on biomass yield (*P<0*.*0001*) compared to cold nutrient enriched biochar, non-enriched biochar and fertilized control treatments.

### Interaction of kiln type and nutrient enrichment of biochar

The interaction of two factors: kiln type and mineral nutrient enrichment type for both biochar dosages showed significant effects (*P<0*.*001*) on maize biomass production (Table 2, bottom row). 1% biochar hot nutrient enriched produced from flame curtain deep cone metal kiln and traditional brick kiln showed higher biomass yield (*P<0*.*001)* compared with 1% non-enriched biochar produced from flame curtain deep cone metal kiln, traditional brick kiln, steel small cone kiln and TLUD (Fig 2, Fig G in S1 File). In contrast, 1% cold nutrient enriched biochar did not show significant effect with 1% non-enriched biochar on maize biomass yield. Furthermore, there was no significant difference between 4% biochar (non-enriched) produced from various kilns and 4% cold nutrient enriched biochar but a significant difference on biomass yield was observed between 4% non-enriched biochar versus 4% hot nutrient enriched biochar produced from flame curtain deep cone metal kiln and traditional brick kiln (Fig 2, Fig G in S1 File).

## Discussion

### Biochar and its effect on soil properties

In this study, the chemical properties of pure biochar produced from "*Eupatorium adenophorum*" via flame curtain kilns were in line with those reported by Schmidt et al, 2015 [28] that used the same feedstock and kiln type biochars qualifying for premium quality of the European Biochar Certificate (EBC). This was the first study where the agronomic effect of biochar produced by flame curtain kilns was compared to that produced via other kilns (traditional brick kiln, TLUD and earth mound kiln). Alkaline biochar (pH 9), when applied to acidic soil, was shown to improve soil chemical properties (pH, CEC and SOC) and reduce deleterious available Al concentration (Table C in S1 File). Increases of soil pH, CEC and SOC were in line with results from earlier studies on sandy and/or acidic soils [40–42].

For SOC, a lower increase was observed than that expected on the basis of additivity (i.e., the amount of C added via the two biochar dosages). This may be due to one or several of these four reasons; i) heterogeneity in soil samples; ii) oxidation of biochar C; iii) leaching of soil or biochar dissolved organic carbon (DOC) [43], or iv) leaching of microscopic biochar particles [44]. Mechanisms (ii) and (iv) were favored by the green house conditions (high temperature and daily irrigation); mechanism (iii) was favored by the increase in alkalinity leading to DOC losses [43]. Biochar is commonly touted for its ability to sequester organic carbon (low C mineralization) for several years [45], however, temperature and moisture availability greatly affects the SOC retention and losses [46]. Biochar stability (CO_2_ sequestered over a 100 year perspective) estimated from literature H/C ratios [47] for the biochar produced from various kiln reported 78% (earth mound kilns), 77% (retort kilns) and 90% (flame curtain kilns, TLUDs, gasifiers) in accordance with differences in operation temperature being lower for earth-mound kiln and retort kilns than for other three kiln types (Table F in S1 File). Thus, freshly produced biochar is not a completely inert material and part of it is prone to oxidation in contact with soil [48]. For example, Hamer et al. reported that C losses depends on feedstock biomass type where biochar produced from corn stover and rye was decomposed more quickly than wood [49].

### Kiln type biochar and its effect on maize biomass production

In this study, the maize biomass production obtained with amendments with biochar made by flame curtain kilns was not shown to be significant different from maize biomass with biochar made with the other kiln types (Table 2, Fig D in S1 File), either non-enriched or enriched. This falsified hypothesis (1), and was corroborated by the observation that kiln type did not result in significant variation in biochar characteristics such as CEC, pH and OC content [50]. Even though flame curtain kilns showed lower emission factors and higher biochar production efficiencies [20], and are operated at higher temperatures, none of the four different flame curtain kilns showed biochar chemical properties (Table A in S1 File) and crop biomass production (Fig D in S1 File) that significantly differed from those observed for biochar generated by the other kilns. In accordance with this, Deal et al [50] reported no variation in biochar characteristics (pH, CEC and OC) produced from different kiln types/pyrolysis temperatures.

Similar non-significant trends of crop yield with kiln type (different pyrolysis conditions) were observed for the biochar produced from ponderosa pine and macadamia nut feedstock under slow and fast pyrolysis types for perennial grass, *Koeleria macrantha* [51] and lettuce/maize corn [52], respectively. Furthermore, biochar produced from traditional kiln type (slow pyrolysis) with rice husk did not show significant effects on rice yield [53].

So far, there have not been any studies that have compared the agronomic effect of biochar produced by various kiln types. Further research on the influence of kiln type on biochar effectiveness for soil and crop yield is thus needed [54]. Soil quality and crop responses generally depend on biochar properties that in turn depend on pyrolysis temperature [55]. Biochar produced from both low and high temperature pyrolysis has shown improvement of soil chemical properties [6,9,40], however, these effects differ greatly dependent on soil mineralogy and types [56]. Without directly comparing kiln types in the same study, crop production in response to biochar produced from different kiln types operated at different temperatures has shown a wide range of effects, from positive to no differences or even negative yield effects [55]. In accordance with our findings for acid soils, meta-analysis showed that increases in crop yield upon biochar amendment were larger for acid soils than for neutral ones (26). However, in contrast to our findings, the authors reported a large variation with biochar properties and, implicitly, kiln types.

### Nutrient enrichment of biochar and its effect on maize biomass

In order to investigate appropriate techniques of mineral nutrient enrichment of biochar, a pot trial was conducted where hot and cold biochar were enriched with liquid mineral fertilizer or applied separately with mineral fertilizer (non-enriched) in acidic soils, all with the same total amount of fertilizer. Nutrient enrichment could be an effective method to improve soil fertility because nutrients become reversibly trapped in the nano/micropores inside the biochar matrix where water movement is restricted, and act as a slow-release fertilizer, reducing nutrient leaching on low CEC soils [42,57]. This is the first study in which hot and cold nutrient enrichment have been compared. Hot nutrient enrichment showed better effects on crop yield than cold nutrient enrichment or separate addition of biochar and nutrients, confirming hypothesis (2).

An explanation why hot nutrient enrichment was more effective than cold nutrient enrichment can possibly be obtained by analogy with organic compound diffusion through soil and black carbon nanopores. The penetration of nutrients into biochar nanopores is most likely an activated process that probably takes place faster at increased temperatures: retarded nanopore diffusion of organic compounds is a highly activated process with activation enthalpies ranging from 60 to 100 kJ/mol [58]. This implies that the retarded pore diffusion rates, and thus the rates of nanopore penetration, increase by approximately a factor of 2 for each 10°C increase in temperature (58). Thus we speculate that pore penetration in hot biochar (e.g., between 60 and 100°C, the expected temperature range when 100–200°C hot biochar is brought into water) could be 100–10,000 times faster than that at room temperature, analogous with observations for organic molecules in black carbon pores that showed 100 times faster diffusion at 60 C than 20°C [59,60].

More research has to be done to explain the underlying nutrient enrichment mechanisms, including nutrient speciation and location on the microscopic level [61], and their effects on crop production. One of the few studies explicitly studying nutrient enrichment of biochar is by Kammann et al [29] who observed that co-composting of biochar enriched the material with nitrate and phosphate. The captured nitrate was largely protected against leaching and partly plant-available. The authors hypothesized that nitrate-water bonding in micro- and nano-pores was the mechanism of nitrate capture in biochar particles.

On the other hand, there is a significant volume of literature showing the nutrient retention ability of biochar [62]. For example, Ventura et al. [63] showed in a field experiment that NO_3_^−^ leaching was reduced by 75% by the addition of 10 t ha^-1^ biochar, whereas NH_4_^+^ leaching was low and not influenced. Also Laird et al [64] observed that 2% biochar reduced total N and total dissolved P leaching from manure-added nutrients by 11% and 69%, respectively.

With regard to the speciation of N nutrients added to biochar, X-ray Photoelectron Spectroscopy (XPS) analysis and SEM imaging of co-composted biochars indicated the presence of iron oxide compounds and amine-NH_3_ on the surface and pores of the biochars (61). Changes in N functional groups on the biochar surface upon composting indicated sorption and/or reaction with other N species [61].

Based on our study, we suggest not to extinguish the hot biochar by adding NPK solution to it just after pyrolysis, since this would lead to excessive N losses as NH_3_ due to biochar's alkaline reaction (NH_4_^+^ can be deprotonated to NH_3_, upon which gaseous losses of N can occur in combination with excessive temperature (200–400°C) (unpublished field observations). Under field conditions, NH_3_ losses upon the addition of urea solution to hot biochar in flame curtain kilns were observed by a strong ammonia smell. It is recommended to first dissolve the NPK in water to which hot biochar can be added after pyrolysis, when temperatures are between 100 and 200°C. These data confirm the research conducted by Schmidt et al. 2015 [28], where biochar enriched with cattle urine showed significantly increased pumpkin yields, with an increase of 300% and 85% compared with only urine treatment and separate biochar and urine addition, respectively [28].

This study also showed that 1% hot nutrient enriched biochar gave significantly higher maize biomass (*P<0*.*0001*) than all of the 4% biochar treatments (hot nutrient enrichment, cold nutrient enrichment and non-enrichment) (Fig 3). This may be due to the fact that the addition of 4% biochar (corresponding to 80 t ha^-1^), is a too high dosage, as has been observed before [41]. The amendment of 4% biochar is perhaps not realistic from a field perspective either and may result in too large alterations in other soil properties (physical, biological).

## Conclusion

Biochar can be produced from the invasive plant species *“Eupatorium adenophorum*” using various different types of kilns. Among all kilns tested, flame curtain kilns showed the lowest gas emissions factors [20], however, the resulting biochar was observed to possess chemical properties and agronomic effect similar to those seen for biochars produced by other kiln types. A weathered soil (low pH, % C and CEC) with resulting low crop production was significantly improved resulting in increased maize biomass when biochar was amended to the soil in this greenhouse experiment. Biochar has shown improved soil chemical properties with increased soil pH, CEC, C and Ca/Al ratio in Nepalese acidic soils. The strongest effect was achieved after directly mixing the hot biochar with a nutrient (NPK) solution, rather than adding biochar and nutrients separately. Importantly, differences in agronomic and chemical quality between biochars generated by various technologies were small compared to differences between biochar nutrient enrichment methods.

## Supporting information

S1 File Image A. Kiln types. Overview of kiln types tested in this paper.
Description A. Biochar Production Technology through different kilns.
Table A. Properties of biochar.
Table B. Nitrogen content (NO_3_-N and NH_4_-N) of hot mineral nutrient (urea) enriched biochar substrate.
Table C. Soil properties of biochar amended and control soils.
Table D. Effect of kiln type biochar enriched with and without mineral nutrients on maize biomass after 50d.
Table E. Effect of kiln type on maize biomass yield after 50 d for non-enrichment biochar and enriched biochar.
Table F. Biochar stability calculated from literature H/C–ratios.
Fig A. Effect of 1% biochar dosage and 4% biochar dosages and both fertilized and non-fertilized control on maize dry AGB (g) production.
Fig B: Effect of biochar (BC) amended soils produced from different kiln and enriched and non-enriched in various ways with 1 and 4% dosages (19 levels) vs control treatments (2 levels) on maize height.
Fig C: Effect of biochar (BC) amended soils produced from different kiln and enriched and non-enriched in various ways with 1 and 4% dosages (19 levels) vs control treatments (2 levels) on maize node diameter.
Fig D: Effect of kiln types biochar on maize dry AGB (g) production.
Fig E. Effect of hot and cold mineral nutrient enrichment and non-enrichment type's biochar on maize height.
Fig F. Effect of hot and cold mineral nutrient enrichment and non-enrichment type's biochar on maize node diameter.
Fig G. Effect of Kiln type biochar (1% and 4% dosages) enriched and non-enriched with mineral nutrient on maize biomass production.
(DOCX)
