# Supplementary material for: Biochar from "Kon Tiki" flame curtain and other kilns: Effects of nutrient enrichment and kiln type on crop yield and soil chemistry
Source: PLoS One. 2017 Apr 27;12(4):e0176378. doi: 10.1371/journal.pone.0176378 (PMC5407783; doi:10.1371/journal.pone.0176378)
Supplement: S1 File — Image A. Kiln types. Overview of kiln types tested in this paper. Description A. Biochar Production Technology through different kilns. Table A. Properties of biochar. Table B. Nitrogen content (NO3-N and NH4-N) of hot mineral nutrient (urea) enriched biochar substrate. Table C. Soil properties of biochar amended and control soils. Table D. Effect of kiln type biochar enriched with and without mineral nutrients on maize biomass after 50d. Table E. Effect of kiln type on maize biomass yield after 50 d for non-enrichment biochar and enriched biochar. Table F. Biochar stability calculated from literature H/C–ratios. Fig A. Effect of 1% biochar dosage and 4% biochar dosages and both fertilized and non-fertilized control on maize dry AGB (g) production. Fig B: Effect of biochar (BC) amended soils produced from different kiln and enriched and non-enriched in various ways with 1 and 4% dosages (19 levels) vs control treatments (2 levels) on maize height. Fig C: Effect of biochar (BC) amended soils produced from different kiln and enriched and non-enriched in various ways with 1 and 4% dosages (19 levels) vs control treatments (2 levels) on maize node diameter. Fig D: Effect of kiln types biochar on maize dry AGB (g) production. Fig E. Effect of hot and cold mineral nutrient enrichment and non-enrichment type's biochar on maize height. Fig F. Effect of hot and cold mineral nutrient enrichment and non-enrichment type's biochar on maize node diameter. Fig G. Effect of Kiln type biochar (1% and 4% dosages) enriched and non-enriched with mineral nutrient on maize biomass production. (DOCX) [file pone.0176378.s001.docx]

**Supplementary Information (SI)**

**Biochar from "Kon Tiki" flame curtain and other Kilns: Effects of Nutrient Enrichment and Kiln Type on Crop Yield and Soil Chemistry**

*Naba Raj Pandit^1,2^, Jan Mulder^1^ , Sarah Hale^3^, Hans Peter Schmidt^4^, Gerard Cornelissen^1,3,*^*

1) Institute for Environmental Sciences (IMV), Norwegian University of Life sciences (NMBU), Ås, Norway

2) Nepal Agroforestry Foundation (NAF), Koteshwor, Kathmandu, Nepal

3) Norwegian Geotechnical Institute (NGI), Oslo, Norway

4) Ithaka Institute for Carbon Strategies, Ancienne Eglise 9, 1974 Arbaz, Switzerland

**Image A:Biochar production technology (Images 1-7)**

Biochar production technology where Eupatorium was used as the feedstock (images with description)

**
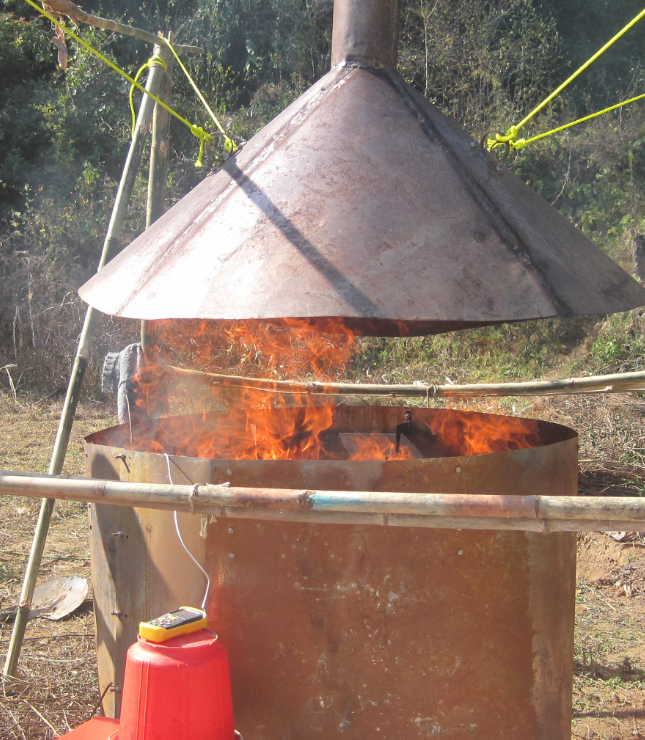
**
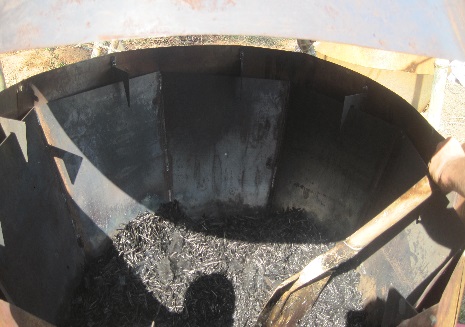

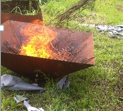


**Image 1:** Flame curtain deep cone metal kiln (left), biochar from flame curtain kiln (middle) and small metal cone kiln (right).

**
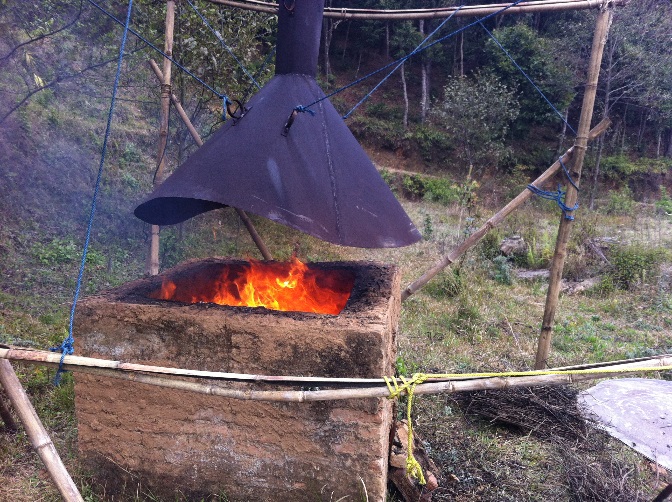

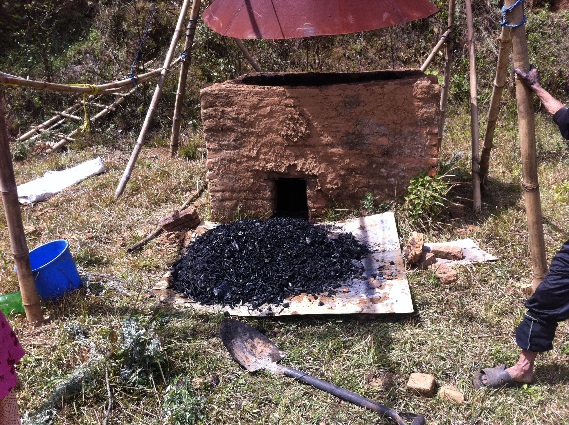
**

**Image 2:** Traditional brick kiln operation (left) and biochar harvested (right). Sizes : Inner wall; 1m x 1m x 1m and Outer wall; 1.4m x 1.4m x 1.4m.


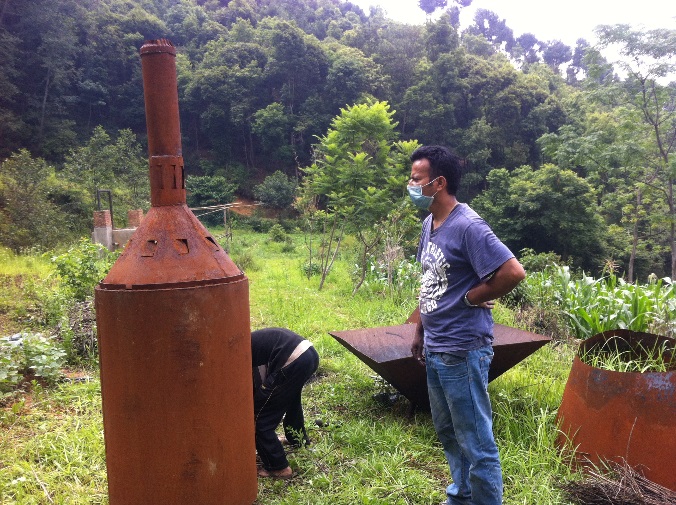

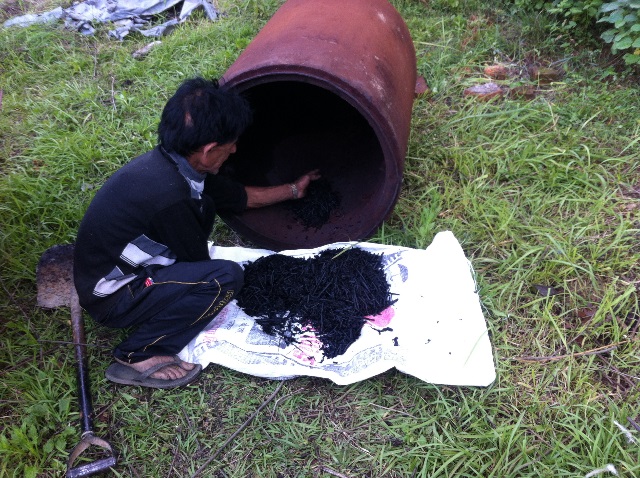


**Image 3:** TLUD operation (left) and biochar harvested (right). Sizes: 0.65 m diameter, 1.05m height and 2.2m circumference.

**
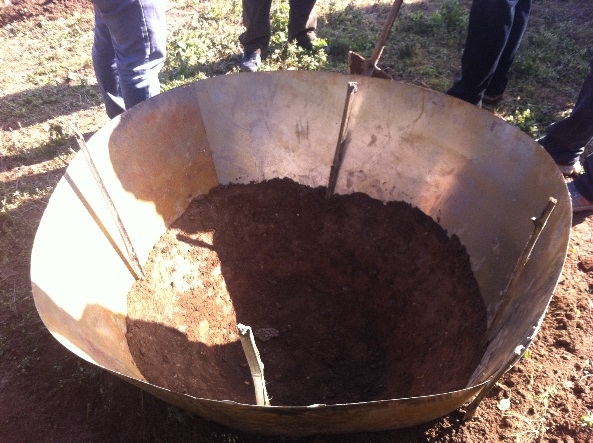

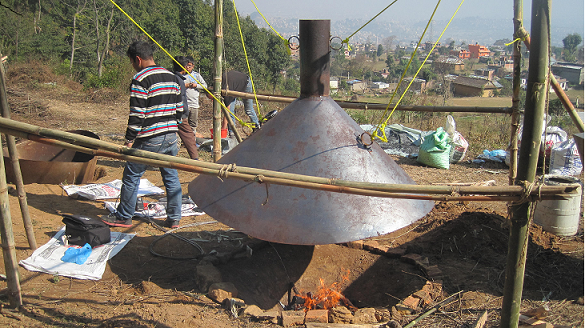
**

**Image 4:** Steel shielded soil pit (left), Sizes: diameter (top: 1.02m, middle: 0.8m and bottom: 0.47m), height: 1.55m, and Conical soil pit (right), Sizes: diameter (top: 0.8m, bottom: 0.47m), height (0.8m).


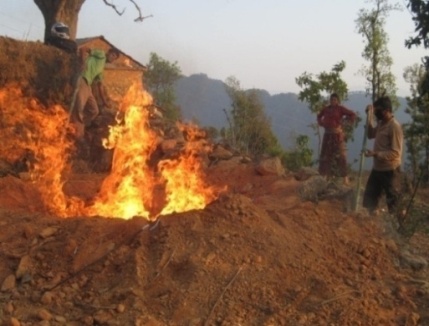

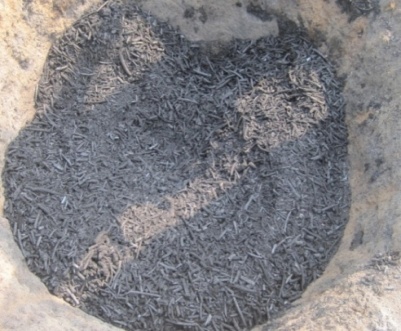


**Image 5:** Traditional earth-mound kiln (1m^3^ dimensions) practiced by farmers in Rasuwa (left) and BC harvested (right)

**
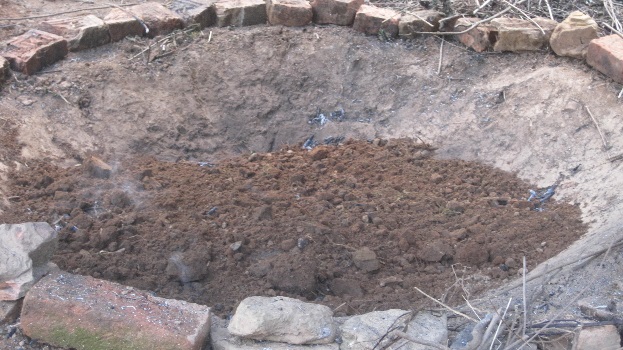

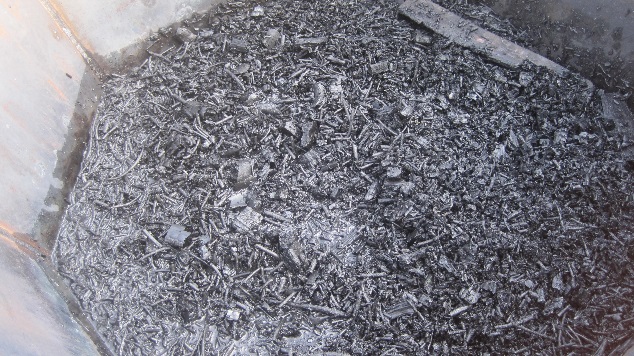
**

**Image 6:** Snuffing with soil (left) and water (right)


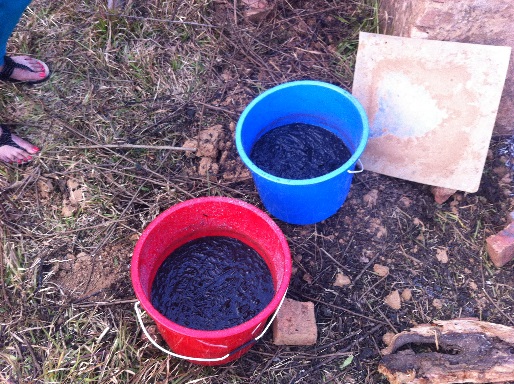
 **
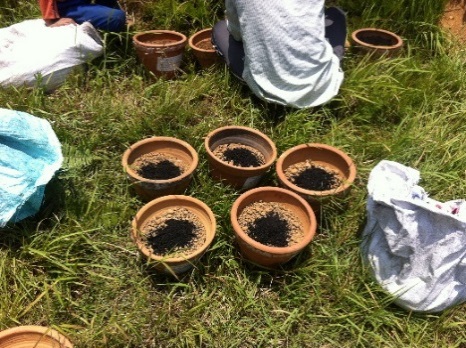
**

**Image 7:** Nutrient enriched biochar (left) and non-enriched biochar before mixing with soil (right)

**Description A. Biochar Production Technology through different kilns**

**Flame curtain deep cone metal kiln and steel small cone Kiln**

The fire was started on the top of the added biomass and the burning sparks ignite the feedstock at the bottom of the metal kiln which form the first layer of biomass. A thin bundle of Eupatorium (loosened) was added on the top of the sparks that heats quickly and starts to outgas. The biomass carbonizes beneath the flames due to low oxygen levels as the oxygen was consumed on the top. The next layer of eupatorium was added homogenously when ash appears on the outside of the carbonizing biomass. The combustion zone establishes a flame curtain that protects the biochar below from oxidizing. Feedstock was added continuously until the metal kiln was filled up and then quenched with water (1–3). The fresh biochar yield was measured immediately after quenching and the sample was collected for biochar dry matter analysis and characterization. For hot mineral nutrient enrichment, the hot biochar just after pyrolysis (700^o^ C) was enriched in dissolved mineral fertilizer (NPK) in the form of urea (5.11 g), di-ammonium phosphate (2.34 g) and potash (1.8 g) containing at the rate of 500ml and 1 L water in the bucket of 10 l capacity for 1% BC and 4% biochar hot nutrient enrichment respectively. Cold nutrient enrichment was carried out in the same way (similar dose of NPK, dimension of a bucket and volume of water) but cooled-down biochar was used instead of still hot biochar.

**Traditional Brick kiln**

The inner and outer walls of the brick kiln are plastered with mud (Image 1). Feedstock (Eupatorium) is placed inside the brick kiln chamber and ignited. Bundles of feedstock are added until the kiln was filled up. After adding the last bundle, the top and bottom hole of the brick kiln are covered with corrugated tin plate for 2 hours without allowing oxygen to gets in. After two hours, the tin plate was removed and biochar was quenched with water and the yield was measured. Biochar sampling and characterization was operated in the same way as kontiki kiln BC.

**Conical soil pit and steel-shielded soil pit**

These kilns work according to the same principle as the flame curtain kiln. The only difference is that flame curtain "kontiki" (deep cone metal kiln and steel small cone) is made up of metal and conical soil pit and steel shielded soil pit was built under soil (Image 4). Feedstock was added layer after layer in the same way as that of kon-tiki until the hole was filled up and quenched with soil unlike kon-tiki. Biochar was harvested after 24 hours and yield was measured. Samples were collected for characterization.

**TLUD (top lit up draft)**

Eupatorium feedstock was kept inside the TLUD and ignited from the top. After the top portion started burning, the top was closed with the lid (Image 3). Pyrolysis was carried out for 18 minutes after which the lid was opened and quenched with water. Biochar yield was measured after quenching and biochar samples were collected.

**Traditional earth-mound kiln**

This kiln was adopted in Rasuwa district (Nepal) by rural farmers to produce a biochar. In this methods a 1 m^3^ hole was dug and Eupatorium feedstock was burned layer after layer as mentioned for the conical soil pit. After the hole was filled up with added feedstocks layers, the last layer was quenched with soil. Yield was measured and the sample was collected for biochar characterization.

**Table A. Properties of biochar.** Biochar carbon yields, Cation exchange capacity (CEC), pH, surface areas (SA) and total C, H, N contents. Biochar produced from different kiln with 100% *Eupatorium* feedstock was either soil or water snuffed**.**

| **S.no** | **Biochar (Kiln type)** | **Quenching** | **Properties of Biochar** | | | | | | | |  |
| --- | --- | --- | --- | --- | --- | --- | --- | --- | --- | --- | --- |
|  |  |  | **DM**  **Yield (%)** | **pH** | | **CEC**  **(Cmolc/kg)** | **C**  **(%)** | **H**  **(%)** | **N**  **(%)** | **C yield**  **%** | **SA m^2^/g** |
|  |  |  |  | **Water** | **Cacl2** |  |  |  |  |  |  |
| 1. | Traditional Brick kiln | Water | 19 | 9.8 | 9.0 | 176 | - | - | - | - | - |
| 2. | Flame curtain- deep cone metal kiln | Water | 18 | 9.9 | 8.7 | 121 | 77 | - | - | 36 | 84.9 |
| 3. | Flame curtain- All steel small cone |  |  |  |  |  |  |  |  |  |  |
| 3.1 | Pyramid 45 | Water | 21 | 10.1 | 9.4 | 193 | 74.1 | 1.33 | 1.04 | 39 | 215 |
| 3.2 | Pyramid 55 | Water | 17 | 10.0 | 9.0 | 100 | 77.2 | 2.01 | 0.72 | 32 | 72.9 |
| 3.3 | Pyramid 60 | Water | 20 | 10.4 | 9.5 | 83 | 84.1 | 2.22 | 0.68 | 42 | - |
| 3.4 | Pyramid 45 with shield | Water | 27 | 10.1 | 9.5 | 217 | 72.5 | - | - | 49 | - |
| 3.5 | Octagonal 60 | Water | 13 | 10.0 | 8.8 |  | 64.7 | - | - | 23 | - |
| 4 | Flame curtain- Steel-shielded soil pit | Soil | 25 | 10.0 | 8.9 | 121 | 81.2 | - | - | 56 | 35.4 |
|  |  |  |  |  |  |  | - | - | - |  |  |
| 5. | Flame curtain- Conical soil pit | Soil | 18 | 10.4 | 9.2 | 127 | 71.4 | 2.16 | 0.66 | 43 | 74.6 |
|  |  |  |  |  |  |  | - | - | - |  |  |
| 6 | TLUD | Water | 12 | 10.2 | 9.0 | 95 | 63.5 | 1.35 | 0.62 |  | - |
| 7 | Traditional earth-mound kiln | Soil | 21 | 10.4 | 9.3 | 86 | - | - | - |  | - |

**Table B: Nitrogen content (NO_3_-N and NH_4_-N)** **of hot mineral nutrient (urea) enriched biochar substrate.** 5g hot enriched biochar sample extracted in 25ml 2M KCl and rest for 24 minutes for N characterization. 3.8 % N and 2% N was available as urea in 1% and 4 % biochar dosages being hot enriched. 0.5% N content was available in biochar itself; thus, with total nitrogen of 4.3% and 2.5% for 1% and 4% biochar hot enrichment respectively.

| **S.no** | **Biochar dosage hot mineral nutrient enrichment** | **NO_3_-N (mg/kg), n=3** | **NH_4_-N (mg/kg), (n=3)** | **C%** | **H%** | **N%** | **%TS** |
| --- | --- | --- | --- | --- | --- | --- | --- |
| **1** | 1% biochar dosages | 1,08 ± 0,12 | 313 ± 5,77 | 76 | 1,7 | 4,3 | 12 |
| **2** | 4% biochar dosages | 0,81 ± 0,02 | 120 ± 0 | 77 | 1,8 | 2,5 | 13 |

**Table C. Soil properties of biochar amended and control soils.** Biochar blended soils encompassed different kilns biochar, mineral nutrient enrichment and non-enrichment biochar that were applied in two (1% and 4% biochar) different dosages (n=19). Two additional control treatments (fertilized and non-fertilized, n=2).

| **Treatments** | | **Kiln type biochar** | **Treatment (enrichment type)** | **pH** | | **Ca/Al** | **CEC** | **Total CHN %** | | | |
| --- | --- | --- | --- | --- | --- | --- | --- | --- | --- | --- | --- |
|  |  |  |  | Water | CaCl_2_ |  | cmolc/kg | | C (%) | H (%) | N (%) |
|  | Traditional Brick kiln biochar | | 1% BC hot mineral nutrient enrichment | 4,82 | 4,42 | 9,6 | 16,35 | | 2,10 | 0,50 | 0,14 |
| **Biochar amended soils** |  |  | 4% BC hot mineral nutrient enrichment) | 6,26 | 5,89 | 1000 | 45,66 | | 3,65 | 0,47 | 0,18 |
|  |  |  | 1% BC cold mineral nutrient enrichment | 4,28 | 4,05 | 3,6 | 16,81 | | 1,60 | 0,48 | 0,41 |
|  |  |  | 4% BC cold mineral nutrient enrichment | 4,69 | 4,59 | 65,2 | 35,29 | | 2,98 | 0,49 | 0,19 |
|  |  |  | 1% BC non-enriched | 4,38 | 4,1 | 4,1 | 17,27 | | 1,81 | 0,47 | 0,15 |
|  |  |  | 4% BC non-enriched | 5,55 | 5,32 | 12,6 | 18,89 | | 3,40 | 0,49 | 0,18 |
|  | Flame curtain Deep cone metal  kiln biochar | | 1% BC hot mineral nutrient enrichment | 4,4 | 4,16 | 7,4 | 16,94 | | 1,97 | 0,49 | 0,23 |
|  |  |  | 4% BC hot mineral nutrient enrichment | 4,48 | 4,32 | 28,6 | 28,41 | | 3,17 | 0,49 | 0,21 |
|  |  |  | 1% BC cold mineral nutrient enrichment | 4,47 | 4,18 | 7,2 | 17,12 | | 2,00 | 0,49 | 0,16 |
|  |  |  | 4% BC cold mineral nutrient enrichment | 5,17 | 4,87 | 100 | 22,44 | | 3,02 | 0,50 | 0,18 |
|  |  |  | 1% BC non-enriched | 4,25 | 4,04 | 4,5 | 16,47 | | 1,93 | 0,47 | 0,16 |
|  |  |  | 4% BC non-enriched | 4,67 | 4,48 | 23,3 | 23,98 | | 3,65 | 0,51 | 0,19 |
|  | Steel small cone biochar | | 1% BC non-enriched | 4,46 | 4,17 | 7,1 | 17,84 | | 1,87 | 0,47 | 0,15 |
|  |  |  | 4% BC non-enriched | 5,47 | 5,28 | 311,1 | 26,18 | | 3,85 | 0,50 | 0,18 |
|  | TLUD BC | | 1% BC non-enriched | 4,33 | 4,06 | 5,7 | 16,95 | | 1,92 | 0,50 | 0,14 |
|  |  |  | 4% BC non-enriched | 4,45 | 4,32 | 14,9 | 21,31 | | 3,68 | 0,53 | 0,22 |
|  | Traditional earth-mound kiln biochar | | 4% BC non-enriched | 4,28 | 4,13 | 6,5 | 21,04 | | 2,85 | 0,47 | 0,17 |
|  | Steel shielded soil pit biochar | | 4% BC non-enriched | 4,62 | 4,39 | 447.3 | 37,18 | | 2,71 | 0,49 | 0,17 |
|  | Conical soil pit biochar | | 4% BC non-enriched | 4,71 | 4,51 | 30,9 | 25,4 | | 2,79 | 0,50 | 0,18 |
| **Control soils** | Control (C1) | | Non-fertilized control | 4,57 | 4,19 | 3,2 | 11,19 | | 1,53 | 0,49 | **0,13** |
|  | Control (C2) | | Fertilized control | 4,34 | 4,01 | 2,4 | 12,11 | | 1,49 | 0,48 | **0,22** |

**Table D: Effect of kiln type biochar enriched with and without mineral nutrients (19 levels) and control treatments (2 levels) on maize biomass after 50 d.** Two factor ANOVA (kiln type and mineral nutrient enrichment biochar and it's interaction) includes 19 levels (T1-T19), N= 77; and one factor ANOVA includes 21 levels (T1-T21), N= 86.

| **ID code**  **Treatment type** | | **Maize Biomass Production** | | | |
| --- | --- | --- | --- | --- | --- |
|  |  | **Height (cm)** | **AGB (g)** | **Node diameter (cm)** | **(n)** |
| T1 | Traditional brick kiln 1% BC hot mineral nutrient enrichment | 124.6 ± 4.72 | 15.84 ± 1.3 | 4.10 ± 0.3 | 5 |
| T2 | Traditional brick kiln 4% BC hot mineral nutrient enrichment | 72.5 ± 5.12 | 7.82 ± 1.1 | 3.10 ± 0.6 | 4 |
| T3 | Traditional brick kiln 1% BC cold mineral nutrient enrichment | 56.8 ± 13.1 | 5.48 ± 1.7 | 2.26 ± 0.7 | 5 |
| T4 | Traditional brick kiln 4% BC cold mineral nutrient enrichment | 58.0 ± 3.6 | 5.33 ± 0.2 | 2.43 ± 0.1 | 3 |
| T5 | Traditional brick kiln 1% BC non-enriched | 53.2 ± 10.7 | 4.12 ± 1.5 | 2.25 ± 0.4 | 4 |
| T6 | Traditional brick kiln 4% BC non-enriched | 51.6 ± 3.2 | 3.76 ± 0.6 | 2.13 ± 0.2 | 3 |
| T7 | Deep cone metal kiln 1% BC hot mineral nutrient enrichment | 107.0 ± 7.1 | 11.94 ± 0.6 | 3.46 ± 0.4 | 5 |
| T8 | Deep cone metal kiln 4% BC hot mineral nutrient enrichment | 84.7 ± 5.6 | 9.92 ± 0.6 | 3.05 ± 0.3 | 4 |
| T9 | Deep cone metal kiln 1% cold mineral nutrient enrichment | 59.7 ± 7.2 | 5.62 ± 0.8 | 2.40 ± 0.4 | 4 |
| T10 | Deep cone metal kiln 4 % cold mineral nutrient enrichment | 54.0 ± 5.3 | 4.64 ± 1.1 | 1.88 ± 0.5 | 5 |
| T11 | Deep cone metal kiln 1% BC non-enriched | 56.0 ± 5.2 | 4.67 ± 0.6 | 1.77 ± 0.3 | 4 |
| T12 | Deep cone metal kiln 4% BC non-enriched | 60.6 ± 5.1 | 5.60 ± 0.5 | 2.20 ± 0.3 | 3 |
| T13 | Steel small cone kiln 1% BC non-enriched | 55.6 ± 5.6 | 4.64 ± 0.7 | 1.74 ± 0.3 | 5 |
| T14 | Steel small cone kiln 4% BC non-enriched | 64.5 ± 9.1 | 5.87 ± 1.1 | 3.46 ± 0.4 | 4 |
| T15 | TLUD 1% BC non-enriched | 48.7 ± 6.2 | 4.37 ± 0.8 | 1.62 ± 0.3 | 4 |
| T16 | TLUD 4% BC non-enriched | 53.7 ± 3.3 | 5.37 ± 0.6 | 1.80 ± 0.4 | 4 |
| T17 | Traditional earth-mound kiln 4% BC non-enriched | 56.6 ± 2.3 | 5.03 ± 0.2 | 2.60 ± 0.3 | 3 |
| T18 | Steel shielded soil pit 4% BC non-enriched | 51.0 ± 4.5 | 3.90 ± 0.8 | 1.56 ± 0.3 | 3 |
| T19 | Conical soil pit 4% BC non-enriched | 54.4 ± 10.5 | 4.88 ± 1.2 | 1.94 ± 0.5 | 5 |
|  |  |  |  |  | ***N = 77*** |
| T20 | Non-fertilized Control (C1) | 44.2 ± 4.6 | 3.02 ± 0.2 | 1.25 ± 0.2 | 4 |
| T21 | Fertilized control (C2) | 51.2 ± 4.6 | 4.44 ± 0.7 | 1.94 ± 0.2 | 5 |
|  |  |  |  |  | ***N= 86*** |
|  |  |  |  |  |  |

**Table E. Effect of kiln type on maize biomass production after 50 d for non-enrichment biochar (N=42) and enriched biochar (N=35)**

| 1. **Non-enrichment biochar (kiln types)** | | | | |
| --- | --- | --- | --- | --- |
| **Kiln types** | **(n)** | **Average Biomass Production** | | |
|  |  | **Maize height** | **AGB** | **Node diameter** |
| Traditional brick kiln | 7 | 52.5 ± 7.8 | 4.2 ± 1.0 | 2.2 ± 0.3 |
| Deep cone metal kiln | 7 | 58.0 ± 5.4 | 5.0 ± 0.7 | 1.9 ± 0.3 |
| Small cone kiln | 9 | 58.5 ± 8.4 | 5.0 ± 1.0 | 1.9 ± 0.4 |
| TLUD | 8 | 51.9 ± 5.4 | 4.8 ± 0.8 | 1.7 ± 0.3 |
| Conical soil pit | 5 | 54.4 ± 10.5 | 4.8 ± 1.2 | 1.9 ± 0.5 |
| Steel shielded soil pit | 3 | 51.0 ±4.5 | 4.3 ± 0.3 | 1.5 ± 0.3 |
| Traditional earth mound kiln | 3 | 56.6 ± 2.3 | 5.0 ± 0.2 | 2.6 ± 0.2 |
| **Average kiln type non-enriched biochar** |  | **54.7 ± 6.4** | **4.7 ± 0.7** | **2.0 ± 0.3** |
| 1. **Enrichment biochar (kiln types)** | | | | |
| Traditional brick kiln | 17 | 80.0 ± 30.3 | 9.1 ± 4.7 | 3.2 ± 0.9 |
| Deep cone metal kiln | 18 | 77.0 ± 23.1 | 8.1 ± 3.2 | 2.7 ± 0.7 |
| **Average kiln type (enriched biochar)** |  | **78.5 ±26.5** | **8.6 ± 4.0** | **3.0 ± 0.8** |

Table F. Biochar stability calculated from literature H/C-ratios according to Camps-Arbestain et al (4).

| **Production technology** | **H/C-ratio** | **Stability (100 y)** | **Reference** |
| --- | --- | --- | --- |
| Earth-mound | **0,43 ± 0,15 (n=7)** | 78% | Martinsen et al. [2]  Obia et al. (in prep), Cornelissen et al. [3] Kupryianchyk et al. [4] |
| Retort | **0,44 ± 0,56 (n=2)** | 77% | Kupryianchyk et al. [4] |
| Flame curtain | **0,22 ± 0,07 (n=14)** | 90% | Schmidt et al. [5, 6] and in prep. |
| Gasifier/TLUD^1^ | **0,28 ± 0,21 (n=4)** | 90% | Shackley et al. [7] |

^1^ **The TLUD stove was assumed to give biochar with same stability as produced with a gasifier as the operation principle is the same**

**Fig. A**: **Effect of 1% biochar dosage (n=43) and 4 % biochar dosages (n=46) and control soils (fertilized control; n=5 and non-fertilized control; n=4) on maize dry AGB (g) yield**. Average maize yield data (1% and 4% biochar dosages) were pooled from biochar produced from different kilns and enriched in various ways. Mean maize AGB yield (g) plotted in y-axis in response to biochar amended (1% and 4% biochar) and control soils on x-axis. Mean AGB yield (g pot^-1^) along with error bars and their respective significance codes (a, b, c) were provided above each bar based on one way ANOVA and post hoc tukey test at 0.05 significance level.

*.*


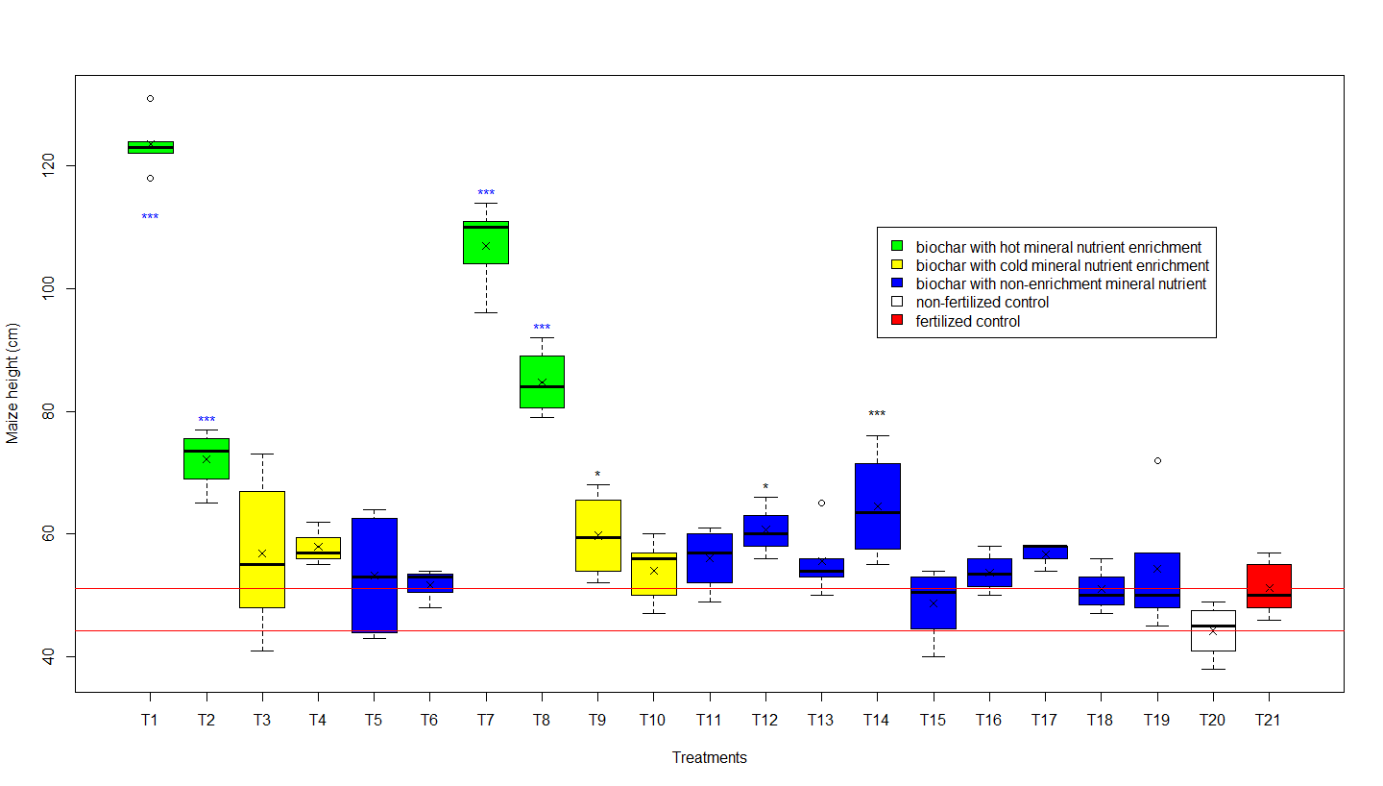


**Fig B:** **Effect of biochar (BC) amended soils produced from different kiln and enriched and non-enriched in various ways with 1 and 4% dosages (19 levels) vs control treatments (2 levels) on maize height.** Description of treatments code (T1, T2,…T21) is mentioned in the table 1 and S4. Sign (x) in the middle of the box plot refer to the average maize AGB of each treatments. Asterisk (*) at the top of the box plot denotes the significant difference between biochar treatments over control (C1/T20 for non-fertilized control and C2/T21 for fertilized control) treatments (*** < 0.001, ** <0.01 and * <0.05 significance) based on one way ANOVA (*levels = 21, N=86 and P < 0.0001)* followed by post hoc tukey test (P <0.05). Blue color asterisk (*) represents significance level for both C1/T20 (no color) and C2/T21 (red color box plot) whereas black color (*) only for C1/T20.

**
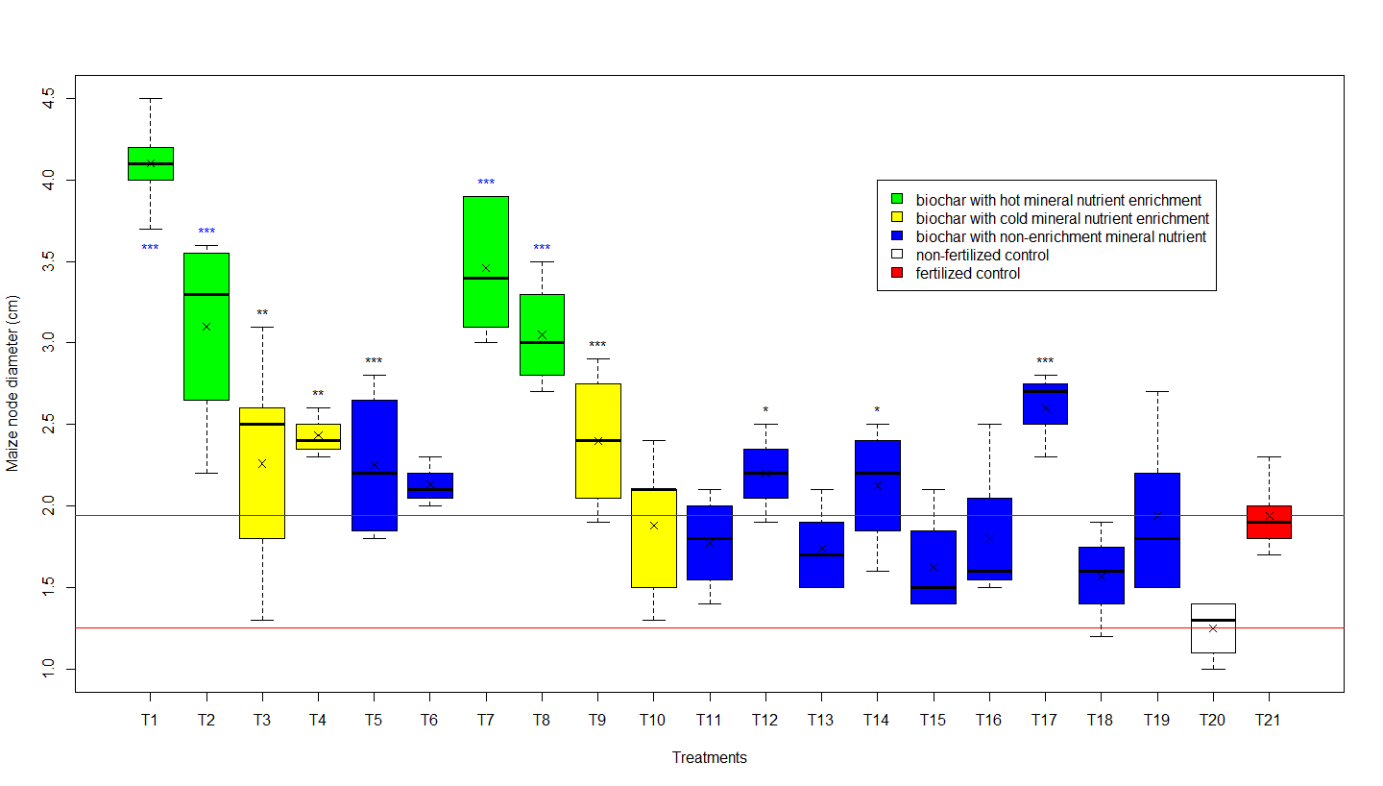
**

**Fig C:** **Effect of biochar (BC) amended soils produced from different kiln and enriched and non-enriched in various ways with 1 and 4% dosages (19 levels, T1, T2..T19) vs control treatments (2 levels, T20 and T21) on maize node diameter.** Description of treatments (T1,T2,…T21) is mentioned in the table 1 and S4. Sign (x) in the middle of the box plot refer to the average maize AGB of each treatments. Asterisk (*) at the top of the box plot denotes the significant difference between biochar treatments over control (C1/T20 for non-fertilized control and C2/T21 for fertilized control) treatments (*** < 0.001, ** <0.01 and * <0.05 significance) based on one way ANOVA (*levels = 21, N=86 and P < 0.0001)* followed by post hoc tukey test (P <0.05). Blue color asterisk represents significance level for both C1/T20 (no color) and C2/T21 (red color box plot) whereas black color (*) only for C1.

**Fig D. Effect of kiln types biochar on maize dry AGB (g) yield.** Mean maize AGB yield (g) along with their error bars plotted in y-axis in response to kiln type's biochar for non-enriched (left) and enriched biochar (right) on x-axis. Letters (a) above the bars represents significance level on maize AGB as a function of kiln types biochar following two way ANOVA (N=77).

**Fig*.* E*.* Effect of hot and cold mineral nutrient enrichment and non-enrichment type's biochar on maize height**. Maize height (cm) is plotted against y-axis and mineral nutrient enrichment techniques along with controls in x-axis. Different letters above the bars (a, b, c) represent significant differences between mineral nutrient enrichment types following two factor ANOVA (N= 77) and post hoc tukey test at 0.05 significance level.

**Fig*.* F. Effect of hot and cold mineral nutrient enrichment and non-enrichment type's biochar on maize node diameter**. Maize node diameter (cm) is plotted against y-axis and mineral nutrient enrichment techniques in x-axis. Different letters above the bars (a, b, c) represent significant differences between mineral nutrient enrichment types following two factor ANOVA (N= 77) and post hoc tukey test at 0.05 significance level.

**Fig. G. Effect of Kiln type biochar (1% and 4% dosages) enriched and non-enriched with mineral nutrient on maize biomass production.** Mean maize AGB yield (g) plotted in y-axis in response to the mineral nutrient enriched and non-enriched biochar produced from different kiln along with controls on x-axis. Different letters above the bars (a, b, c) represent significant differences between the treatments following two factor ANOVA (N= 77) and post hoc tukey test (P< 0.05).

**References**

1. Cornelissen G, Pandit NR, Taylor P, Pandit BH, Sparrevik M, Schmidt HP. Emissions and Char Quality of Flame-Curtain“ Kon Tiki” Kilns for Farmer-Scale Charcoal/Biochar Production. PLoS One. 2016;11(5):e0154617.

2. Schmidt HP, Taylor P. Kon-Tiki flame curtain pyrolysis for the democratization of biochar production. Biochar J. 2014;1:14–24.

3. Schmidt H, Pandit B, Martinsen V, Cornelissen G, Conte P, Kammann C. Fourfold Increase in Pumpkin Yield in Response to Low-Dosage Root Zone Application of Urine-Enhanced Biochar to a Fertile Tropical Soil. Agriculture [Internet]. 2015 Sep 7 [cited 2015 Sep 20];5(3):723–41. Available from: http://www.mdpi.com/2077-0472/5/3/723/htm

4. Camps-Arbestain M, Amonette JE, Singh B, Wang T, Schmidt HP. A biochar classification system and associated test methods. Biochar Environ Manag Sci Technol Implement. 2015;165–93.
